# 基于上海郊区自然人群队列的豆制品摄入与肺癌发病风险的前瞻性队列研究

**DOI:** 10.3779/j.issn.1009-3419.2025.106.09

**Published:** 2025-04-20

**Authors:** Shiyun DING, Wenhui WU, Jianing MAO, Jingrao LI, Ji ZHENG, Ye YAO, Genming ZHAO, Yiling WU, Ruoxin ZHANG

**Affiliations:** ^1^200030 上海，复旦大学公共卫生学院流行病学教研室，公共卫生安全教育部重点实验室（丁诗韵，武文汇，毛嘉宁，李竞娆，郑吉，赵根明，张若昕）; ^1^Department of Epidemiology, School of Public Health, Key Laboratory of Public Health Safety, Ministry of Education,Fudan University, Shanghai 200030, China; ^2^322000 义乌，复旦大学义乌研究院（郑吉，张若昕）; ^2^Yiwu Research Institute, Fudan University, Yiwu 322000, China; ^3^200030 上海，复旦大学公共卫生学院生物统计学教研室（姚烨）; ^3^Department of Biostatistics, School of Public Health, Fudan University, Shanghai 200030, China; ^4^201620 上海，上海市松江区疾病预防控制中心（上海市松江区卫生健康监督所）（吴毅凌）; ^4^Songjiang District Center for Disease Control and Prevention (Shanghai Songjiang District Health Inspection Institute), Shanghai 201620, China

**Keywords:** 肺肿瘤, 豆制品摄入, 多基因风险评分, 肺癌风险, 队列研究, Lung neoplasms, Soy product intake, Polygenic risk score, Lung cancer risk, Cohort study

## Abstract

**背景与目的:**

肺癌是全世界发病率最高的恶性肿瘤之一，探索肺癌发病的影响因素对其防治具有重要意义。尽管生活习惯和遗传因素已被证实与肺癌的发生密切相关，但饮食因素，如豆制品摄入对肺癌发病风险的影响仍不完全明确。本研究旨在探讨豆制品摄入、遗传风险与肺癌发病风险之间的关联，验证豆制品摄入在欧洲人群中的效应一致性，为肺癌防治提供新的思路。

**方法:**

本研究首先基于上海郊区自然人群队列（Shanghai Suburban Adult Cohort and Biobank, SSACB）（n=66,311）使用Cox比例风险回归模型分析豆制品摄入与肺癌发病风险之间的关联，并根据性别、吸烟及肺癌病理类型对人群进行分层分析。采用英国生物银行（UK Biobank, UKB）人群验证豆制品对于肺癌发病的影响。在探究遗传因素与肺癌发病的关联中，在整合既往研究报道的中国人群遗传变异的基础上新增了东南地区两大独立人群的新发现位点进行补充，包括SSACB（433例病例/650例对照）及肿瘤医院-泰州队列（1359例病例/1359例对照）。通过meta分析及连锁不平衡聚类分析（linkage disequilibrium clumping, LD clumping）后，最终筛选出23个显著位点，用于构建多基因风险评分（polygenic risk score, PRS）模型。随后，利用条件Logistic回归模型评估PRS对肺癌发病风险的影响。

**结果:**

在SSACB人群中，调整年龄、性别、吸烟、慢性支气管炎、身体质量指数（body mass index, BMI）、蔬菜及红肉摄入后发现，摄入足量豆制品与肺癌发病风险降低有显著关联[风险比（hazard ratio, HR）=0.60，95%CI: 0.47-0.77，P_adj_=6.69E-05]，且该效应在男性、女性、吸烟和非吸烟人群中均保持一致。在UKB人群中豆制品与肺癌发病的关系虽然没有统计学差异，但也呈现出对肺癌的保护趋势（HR=0.76, 95% CI: 0.55-1.06, P_adj_=0.10）。在SSACB的巢式病例对照人群中，基于中国人群构建的PRS评分与肺癌发病显著相关，调整年龄、性别、吸烟、慢性支气管炎及豆制品摄入后，与低风险人群相比，高PRS评分人群的发病风险是低评分人群的1.88倍（P_adj_=1.84E-03）。

**结论:**

通过前瞻性队列研究发现，摄入足量豆制品可显著降低肺癌发病风险，而高PRS是肺癌发病的重要危险因素。将豆制品摄入量和PRS作为肺癌发病的影响因素进行综合评估，可为肺癌高危人群的个体化分层和精准预防提供一定指导作用。

## Abstract (en)

**Background and objective:**

Lung cancer is one of the malignant cancers with the highest incidence rate, and it is important to identify the factors contributing to lung cancer carcinogenesis for prevention. Lifestyle and genetic factors play important roles in cancer development, however the impact of dietary factors, such as soy product intake, on lung cancer risk remains inadequately understood. This study aims to explore the associations between soy product intake, genetic risk, and lung cancer incidence, and validate the consistent effects of soy product intake in European populations, thereby providing new insights for lung cancer prevention.

**Methods:**

Utilizing the Shanghai Suburban Adult Cohort and Biobank (SSACB) (n=66,311), Cox proportional hazards model was adopted to assess the association between soy product intake and lung cancer incidents, followed by subgroup analyses stratified by gender, smoking status, and pathological types of lung cancer. The UK Biobank (UKB) was used for validation of the effect of soy product intake on lung cancer. To investigate the association between genetic factors and lung cancer, in addition to previously reported loci, we incorporated newly identified loci from two independent studies in Southeast China: a nested case-control population from the SSACB cohort (433 cases/650 controls) and a case-control study from the Shanghai Cancer Center-Taizhou cohort (1359 cases/1359 controls). Meta-analysis and Linkage disequilibrium clumping (LD clumping) of the association results identified 23 loci for polygenic risk score (PRS) construction. Subsequently, conditional Logistic regression model was used to assess the association between genetic risk and lung cancer.

**Results:**

In SSACB cohort, after adjusting for age, gender, smoking, chronic bronchitis, body mass index (BMI), vegetable intake and red meat intake, sufficient soy product intake was significantly associated with a reduced risk of lung cancer [hazard ratio (HR)=0.60, 95%CI: 0.47-0.77, P_adj_=6.69E-05], an effect that was consistent in males and females, smokers and non-smokers. In UKB, although the association did not reach statistical significance, a protective trend against lung cancer was also observed (HR=0.76, 95%CI: 0.55-1.06, P_adj_=0.10). In the nested case-control population within SSACB, a PRS score generated in the Chinese population was significantly correlated with lung cancer risk. After adjustment of age, gender, smoking, chronic bronchitis, and soy product intake, the high-PRS group had a 1.88 times higher risk of lung cancer compared to the low-PRS group (P_adj_=1.84E-03).

**Conclusion:**

The prospective cohort study found that adequate intake of soy products was significantly associated with a reduced risk of lung cancer, while a high PRS is a risk factor for lung cancer development. Integrating soy product intake and PRS into traditional epidemiological risk factor prediction will guide personalized lung cancer prevention and high-risk population stratification.

肺癌的发病率在全球一直居高不下，2022年其发病率和死亡率均在世界范围内位列第一^[1]^。在西方国家肺癌的发病率有所下降，然而中国其发病率仍在上升^[2]^。2022年中国的肺癌标化发病率为40.8/100,000，死亡率也位居肿瘤之首^[1]^。

肺癌的发生是一个长期的过程，来自于环境和遗传的共同作用^[3]^。环境因素方面，吸烟是肺癌的主要危险因素^[3]^，其他常见的因素包括饮食、空气污染、职业暴露等 ^[4-6]^。随着健康生活观念的增强，饮食再次成为人们关注的重点。作为东亚国家日常饮食的重要组成部分之一，以大豆为主的豆制品摄入被多项研究^[7-9]^报道与肺癌风险降低有关，然而相关研究数量有限，并且缺乏在中国人群中的前瞻性研究。

除了环境和生活习惯因素之外，遗传因素也是肺癌发生的重要影响因素。至今为止，全基因组关联分析（genome-wide association study, GWAS）是发现肺癌相关易感基因的重要方法。然而，单个研究发现的易感性遗传位点并不能充分代表个体的患癌风险，使其推广性受到限制。多基因风险评分（polygenic risk score, PRS）被作为量化个体遗传易感性的有效方法，被认为能更全面地实现对个体肺癌风险的预测^[10]^。

本研究基于上海郊区自然人群队列（Shanghai Suburban Adult Cohort and Biobank, SSACB），系统探讨了基线时期豆制品摄入与新发肺癌之间的剂量-反应关系，并在英国生物银行（UK Biobank, UKB）的欧洲人群数据中进行外部验证。此外，对队列的巢式病例对照人群进行GWAS芯片检测，构建出针对中国人群的PRS模型，并探索遗传因素对肺癌发病风险的影响。本研究旨在揭示生活习惯因素和遗传因素对肺癌发病的影响，为肺癌防治和干预提供新的科学依据。

## 1 材料与方法

### 1.1 研究对象

本研究的发现集来自于SSACB，该队列采用多阶段分层整群抽样的方法，在松江、嘉定、闵行三个区招募了69,116名20-74岁的居民加入队列。人员招募及基线资料收集从2016年4月至2017年10月，末次随访时间为2023年7月。队列的建设情况详情参考既往文献^[11]^。新发肺癌患者定义为基线调查3个月之后发病，且通过组织活检等病理检查被确诊为原发性肺癌的患者。健康人群为基线调查至随访截止时未发生恶性肿瘤的人群。在第一部分豆制品摄入与肺癌的关联研究中排除了1403例基本信息和饮食信息缺失的居民、1279例患有其他恶性肿瘤病史以及123例基线前已发病的肺癌患者，最终纳入66,311例进行分析（图1）。在PRS与肺癌关联的研究中，首先汇总了既往在中国人群中的肺癌GWAS所报道的基因位点^[12]^。除此之外采用了两个病例对照人群：第一个巢式病例对照人群来自SSACB队列，共纳入433例肺癌患者，并随机抽取1473例健康对照，按照性别、年龄与对照进行1:1.5匹配，最终纳入433例病例和650例对照；第二个人群为来自于复旦大学上海肿瘤医院（Fudan University Shanghai Cancer Center, FUSCC）和泰州纵向队列（Taizhou Longitudinal Study, TZL）的病例对照人群（FUSCC-TZL），其中肺癌病例为2009至2011年确诊的原发性肺癌患者（具体信息详见文献报道^[13]^）。通过性别和年龄1:1匹配，最终纳入1359例病例和1359例对照（图1）。

**图1:**
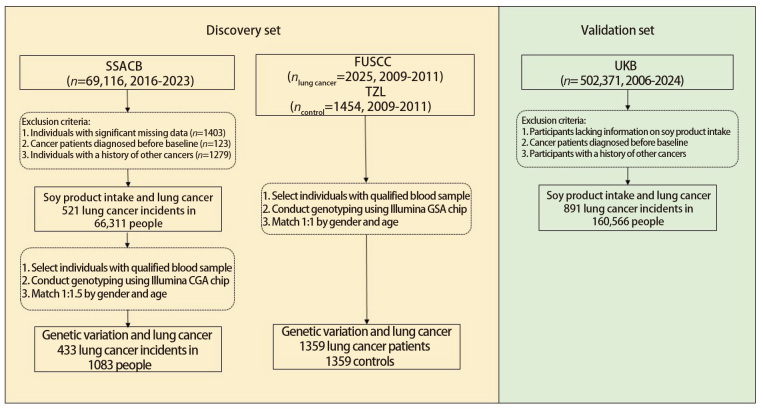
研究对象筛选流程图

验证集来自UKB中有豆制品摄入信息的子队列人群，肺癌患者为2006至2024年新诊断并经病理活检证实为原发性肺癌的人群。在排除豆制品、蔬菜及红肉摄入信息缺失的人群后，最终纳入160,566例进行分析。本研究的研究对象签署知情同意书，且研究方案经复旦大学公共卫生学院医学研究伦理委员会审核通过（IRB No.2016-04-0586）。

### 1.2 信息收集

本研究收集的资料来自于基线问卷调查信息、人体测量数据信息和GWAS芯片检测。基线问卷调查中与本研究相关的内容包括：（1）人口学资料：年龄、性别、受教育程度、职业、婚姻状况、经济状况等；（2）生活方式资料：吸烟、饮酒、喝茶、运动、睡眠障碍情况；（3）膳食情况资料：基于食物频率问卷（Food Frequency Ques-tionnaire, FFQ）的蔬菜、水果、肉类、豆制品等食物摄入种类、摄入量及摄入频率；（4）既往病史及疾病家族史资料，如恶性肿瘤等。在人体测量数据和生物样本采集方面，所有研究对象均被邀请到当地社区卫生服务中心接受免费体检，内容包括身高、体重测量及血液样本采集。

### 1.3 膳食评估

本研究膳食评价建立在FFQ基础之上，根据过去12个月内进食的食物频率和每次平均食用量进行评估。其中，豆制品摄入的类别主要包括豆浆（豆奶）及豆腐。

根据中国居民平衡膳食宝塔（2022年）^[14]^，本研究中将人群的膳食摄入量分为三大类（对应本研究所用到的饮食摄入量）：（1）豆制品摄入量（≤25 g/d为少量，25 g/d<摄入量≤35 g/d为适量，>35g/d为足量）；（2）蔬菜类摄入量（≤300 g/d为少量，300 g/d<摄入量≤500 g/d为适量，>500 g/d 为足量）；（3）红肉类摄入量（≤40 g/d为少量，40 g/d<摄入量≤75 g/d为适量，>75 g/d为过量）。

UKB的饮食调查和中国人群差异较大，由于豆制品的摄入量普遍低于中国人群，根据当地饮食习惯，将豆制品摄入分为3类（0 g/d为少量，0 g/d<摄入量≤35 g/d为适量，>35 g/d为足量）。蔬菜及红肉的每日摄入量按三分位数分为3类。

采用Tukey法排除膳食摄入量中的异常值，以四分位数间距（interquartile range, IQR）为基准，将超出1.5倍IQR范围的数值作为异常值并予以删除。

### 1.4 膳食因素的共线性分析及限制性立方样条图

本研究中各类膳食摄入均纳入共线性分析的范畴，方差膨胀因子（variance inflation factor, VIF）用于衡量多重共线性，如果 VIF=1，表示变量之间完全独立，若VIF>5则被视为中度共线性。结合限制性立方样条法分析豆制品摄入量连续变化与肺癌发病风险之间的剂量-反应关系。以膳食平衡宝塔中豆制品的推荐摄入量（25-35 g/d）为阈值，使用ggplot2包绘制限制性立方样条图。

### 1.5 GWAS芯片数据分析、质量控制和填补

通过收集研究对象的外周血样本提取基因组DNA，基因分型采用Illumina公司核心全局阵列芯片（core global array, CGA）及全球筛查阵列芯片（global screening array, GSA）进行。对基因分型数据进行以下质量控制：（1）排除重复位点；（2）剔除缺失率大于5%的位点；（3）去除最小等位基因频率（minor allele frequency, MAF）小于5%的位点；（4）排除定位于性染色体上的位点；（5）剔除不符合哈迪-温伯格平衡（P<1E-07）的位点。经过质控后，FUSCC-TZL人群共保留357,438个位点，SSACB队列人群保留471,718个位点。

通过西湖大学填补服务器（https://imputationserver.westlake.edu.cn/）进行全基因组填补，以西湖生物样本库（Westlake BioBank for Chinese, WBBC）联合东亚人群（East Asian, EAS）作为参照，通过填补，两个芯片的位点重合率得到大幅度提高。填补后FUSCC-TZL和SSACB的位点数分别为6,992,485和7,036,698个。

### 1.6 PRS模型的构建

用于构建PRS模型的遗传位点由两部分组成。第一部分位点通过GWAS catalog数据库（https://www.ebi.ac.uk/gwas/）以“Lung Cancer”为关键词筛选获得，汇总了中国人群中肺癌GWAS中已报道的位点。根据种族（发现集及验证集均为东亚人群）和显著性水平（P<5E-08）进行初步筛选后，共获得115个与肺癌发生显著相关的位点。其中，10个位点在SSACB人群中达到显著性水平（P<0.05），另有13个位点在中国慢性病前瞻性研究（China Kadoorie Biobank, CKB）中得到验证^[15]^。为确保人群代表性，最终第一部分保留了23个在中国人群队列中与肺癌发生显著相关的位点。

第二部分来自于本研究中国东南部人群的新发现位点。本研究对FUSCC-TZL和SSACB的巢式病例对照人群的GWAS数据进行了meta分析，共得到138个与肺癌发生显著关联的位点，通过与第一部分的23个位点进行连锁不平衡聚类分析（linkage disequilibrium clumping, LD clumping）后，最终筛选出23个独立位点用于PRS的构建（图2）。基于各风险等位基因的数量及其效应值（β值），采用加权回归方法构建PRS模型[PRS=Σ(β_i_×G_i_)]。在条件Logistic回归模型分析中根据PRS的四分位数将人群遗传风险分为低（min-Q1）、中（Q1-Q3）、高（Q3-max）风险组3类。

**图2:**
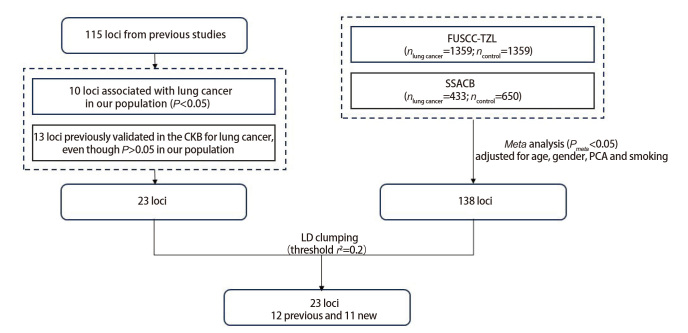
用于PRS构建的遗传位点筛选流程图

### 1.7 统计分析

运用R软件（http://www.R-project.org）进行统计分析。本研究中计量资料均不符合正态分布，使用中位数及四分位数间距表示，采用秩和检验比较组间差异。计数资料以频数和构成比（n, %）表示，采用χ^2^检验比较组间的差异，在样本期望频数T<5的情况下，采用Fisher确切概率法进行组间差异检验。通过Cox比例风险回归模型和条件Logistic回归模型分别分析饮食因素和遗传因素与肺癌发病之间的关系。采用双侧检验，P<0.05为差异有统计学意义。

## 2 结果

### 2.1 人群基线基本特征

随访至2023年7月，SSACB队列共有521例新发肺癌病例，未发生肿瘤者65,790人，平均年龄为59.0（51.0-66.0）岁，其中女性占59.4%。在本队列人群中，豆制品摄入≤25 g/d的个体占比最高（51.6%），其次为豆制品摄入量>35 g/d的人群（29.1%），而摄入适量的人群占比为19.3%（表1）。身体质量指数（body mass index, BMI）、吸烟和慢性支气管炎等指标均在不同豆制品摄入组间存在统计学差异（P<0.05）。此外，教育水平分析显示，高摄入组中大学及以上学历的比例显著更高，表明教育水平可能通过影响健康知识的获取而影响膳食选择行为。

**表1:** SSACB的基线人口学特征

Characteristics	All	Soy intake≤25 g/d	25 g/d<Soy intake≤35 g/d	Soy intake>35 g/d	P
Participants (n, %)	66,311	34,209 (51.6)	12,800 (19.3)	19,302 (29.1)	
Age, median (IQR) (yr)	59.0 (51.0, 66.0)	60.0 (53.0, 66.0)	59.0 (51.0, 65.0)	57.0 (46.0, 65.0)	<2.2E-16
Gender (n, %)					<2.2E-16
Male	26,932 (40.6)	13,052 (38.1)	5431 (42.5)	8449 (43.8)	
Female	39,379 (59.4)	21,157 (61.9)	7369 (57.5)	10,853 (56.2)	
BMI (n, %) (kg/m^2^)					2.27E-11
<18.5	1745 (2.6)	854 (2.5)	344 (2.7)	547 (2.8)	
18.5-24.0	31,071 (46.9)	15,619 (45.7)	6000 (46.9)	9452 (49.0)	
24.1-28.0	25,424 (38.3)	13,346 (39.0)	4990 (39.0)	7088 (36.7)	
>28.0	8071 (12.2)	4390 (12.8)	1466 (11.4)	2215 (11.5)	
Smoking (n, %)					5.67E-07
No	51,662 (77.9)	26,865 (78.5)	9757 (76.2)	15,040 (77.9)	
Yes	14,649 (22.1)	7344 (21.5)	3043 (23.8)	4262 (22.1)	
Chronic bronchitis (n, %)					5.77E-06
No	61,515 (92.8)	31,586 (92.3)	11,887 (92.9)	18,042 (93.5)	
Yes	4796 (7.2)	2623 (7.7)	913 (7.1)	1260 (6.5)	
Education level (n, %)					<2.2E-16
Primary school or below	22,910 (34.5)	15,035 (44.0)	4066 (31.8)	3809 (19.8)	
Junior high school	25,008 (37.7)	12,361 (36.1)	5328 (41.6)	7319 (37.9)	
High school	11,046 (16.7)	4345 (12.7)	2178 (17.0)	4523 (23.4)	
University or above	7347 (11.1)	2468 (7.2)	1228 (9.6)	3651 (18.9)	
Marriage (n, %)					<2.2E-16
Married	60,490 (91.2)	31,223 (91.3)	11,892 (92.9)	17,375 (90.0)	
Unmarried/Divorced	5821 (8.8)	2986 (8.7)	908 (7.1)	1927 (10.0)	

BMI: body mass index; IQR: interquartile range.

验证集UKB中共纳入160,566例具有完整豆制品调查信息的居民，平均年龄为56.0（49.0-62.0）岁，其中女性占54.7%（表2）。在欧洲人群中豆制品摄入量普遍较低，反映出不同地区膳食习惯的差异。

**表2:** UKB队列的基线人口学特征

Characteristics	All	Soy intake=0 g/d	0 g/d<Soy intake≤35 g/d	Soy intake>35 g/d	P
Participants (n, %)	160,566	149,601 (93.2)	2131 (1.3)	8834 (5.5)	
Age, median (IQR) (yr)	56.0 (49.0-62.0)	56.0 (49.0-62.0)	55.0 (48.0-62.0)	56.0 (49.0-62.0)	7.71E-03
Gender (n, %)					0.06
Male	72,679 (45.3)	67,783 (45.3)	911 (42.8)	3985 (45.1)	
Female	87,887 (54.7)	81,818 (54.7)	1220 (57.2)	4849 (54.9)	
BMI (n, %) (kg/m^2^)					0.02
<18.5	870 (0.5)	803 (0.5)	7 (0.3)	60 (0.7)	
18.5-24.0	58,057 (36.2)	53,957 (36.1)	824 (38.7)	3276 (37.1)	
24.1-28.0	67,924 (42.3)	63,399 (42.4)	875 (41.1)	3650 (41.3)	
>28.0	33,715 (21.0)	31,442 (21.0)	425 (19.9)	1848 (20.9)	
Smoking (n, %)					8.84E-01
No	92,529 (57.6)	86,216 (57.5)	1217 (57.0)	5096 (57.6)	
Yes	68,037 (42.4)	63,385 (42.5)	914 (43.0)	3738 (42.4)	
Drinking (n, %)					1.28E-13
No	5497 (3.4)	4980 (3.3)	101 (4.7)	416 (4.7)	
Yes	155,069 (96.6)	144,621 (96.7)	2030 (95.3)	8418 (95.3)	
Chronic bronchitis (n, %)					0.20
No	159,810 (99.5)	148,888 (99.5)	2119 (99.4)	8803 (99.6)	
Yes	756 (0.5)	713 (0.5)	12 (0.6)	31 (0.4)	

### 2.2 豆制品摄入与肺癌发病的关联

单因素Cox比例风险回归分析显示低量蔬菜摄入和低量豆制品摄入均与肺癌发生风险升高显著关联。在矫正了性别、年龄、吸烟、慢性支气管炎、BMI及饮食摄入因素后，蔬菜和豆制品与肺癌的关联仍有统计学意义。其中，足量豆制品摄入与肺癌发生风险降低有明显关联（HR=0.60, 95%CI: 0.47-0.77, P=6.69E-05）（表3）。

**表3:** SSACB饮食因素和新发肺癌的关联

Factors		Univariate model			Multivariate model							
					Model 1			Model 2			Model 3	
		HR (95%CI)	P		HR (95%CI)	P		HR (95%CI)	P		HR (95%CI)	P
Age		1.04 (1.03-1.05)	<2.00E-16		1.04 (1.03-1.05)	2.64E-15		1.04 (1.03-1.05)	4.03E-15		1.04 (1.03-1.05)	2.47E-15
Gender	Male	1.00 (Ref)			1.00 (Ref)			1.00 (Ref)			1.00 (Ref)	
	Female	0.74 (0.62-0.87)	4.46E-04		0.76 (0.64-0.91)	1.70E-03		1.03 (0.80-1.32)	0.84		1.02 (0.79-1.31)	0.90
Smoking	No	1.00 (Ref)						1.00 (Ref)			1.00 (Ref)	
	Yes	1.66 (1.38-1.99)	6.20E-08					1.60 (1.23-2.09)	5.45E-04		1.60 (1.22-2.09)	5.98E-04
Chronic bronchitis	No	1.00 (Ref)						1.00 (Ref)			1.00 (Ref)	
	Yes	1.87 (1.44-2.42)	2.20E-06					1.62 (1.24-2.11)	3.67E-04		1.62 (1.24-2.11)	3.65E-04
BMI	<18.5 kg/m^2^	0.72 (0.40-1.32)	0.29					0.82 (0.45-1.51)	0.53		0.82 (0.45-1.50)	0.52
	18.5-24.0 kg/m^2^	1.00 (Ref)						1.00 (Ref)			1.00 (Ref)	
	24.1-28.0 kg/m^2^	0.88 (0.73-1.06)	0.17					0.79 (0.65-0.96)	0.01		0.79 (0.65-0.96)	0.01
	>28.0 kg/m^2^	0.75 (0.56-1.01)	0.06					0.68 (0.50-0.91)	0.01		0.68 (0.50-0.91)	0.01
Vegetable intake	≤300 g/d	1.00 (Ref)									1.00 (Ref)	
	300-500 g/d	0.55 (0.38-0.80)	1.73E-03								0.53 (0.36-0.79)	1.54E-03
	>500 g/d	0.67 (0.43-1.07)	0.09								0.78 (0.49-1.23)	0.28
Red meat intake	≤40 g/d	1.00 (Ref)									1.00 (Ref)	
	40-75 g/d	0.95 (0.77-1.17)	0.64								1.08 (0.87-1.35)	0.49
	>75 g/d	0.74 (0.57-0.96)	0.03								0.96 (0.74-1.26)	0.78
Soy product intake	≤25 g/d	1.00 (Ref)			1.00 (Ref)			1.00 (Ref)			1.00 (Ref)	
	25-35 g/d	0.91 (0.73-1.13)	0.38		0.94 (0.75-1.17)	0.56		0.93 (0.75-1.17)	0.55		0.94 (0.75-1.18)	0.62
	>35 g/d	0.53 (0.42-0.67)	8.57E-08		0.59 (0.46-0.75)	1.07E-05		0.58 (0.45-0.73)	1.03E-05		0.60 (0.47-0.77)	6.69E-05

Model 1: Adjusted for age and gender; Model 2: Adjusted for age, gender, smoking, chronic bronchitis and BMI; Model 3: Adjusted for Model 2+vegetable intake and red meat intake.

为了评估各饮食因素间的潜在共线性关系，研究使用VIF矩阵判断共线性（表4展示了各饮食变量间的VIF值），结果显示，VIF均接近1，表示饮食变量之间共线性弱，观测的豆制品关联不受到明显的混杂效应影响。

**表4:** SSACB饮食信息VIF值矩阵

Dietary information	Carbohydrate	Vegetable	Fruit	Red meat	Seafood	Soy product	Pickle	Processed meat
Carbohydrate	1.00	1.01	1.00	1.01	1.00	1.00	1.00	1.00
Vegetable	1.01	1.00	1.03	1.04	1.08	1.02	1.00	1.00
Fruit	1.00	1.03	1.00	1.02	1.03	1.01	1.00	1.00
Red meat	1.01	1.04	1.02	1.00	1.09	1.02	1.01	1.03
Seafood	1.00	1.08	1.03	1.09	1.00	1.04	1.00	1.01
Soy product	1.00	1.02	1.01	1.02	1.04	1.00	1.00	1.01
Pickle	1.00	1.00	1.00	1.01	1.00	1.00	1.00	1.02
Processed meat	1.00	1.00	1.00	1.03	1.01	1.01	1.02	1.00

VIF: variance inflation factor.

限制性立方样条法用于分析膳食摄入量连续变化与肺癌发病风险的剂量-反应关系，图3展示了豆制品的连续摄入量与肺癌发病风险之间存在负相关。在本研究人群中豆制品摄入量的增加与肺癌发病风险的降低显著相关。

**图3:**
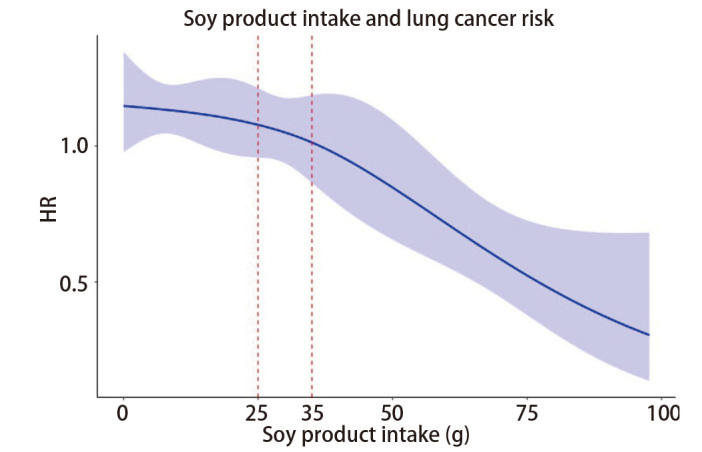
豆制品摄入与肺癌发病风险的限制性样条图

研究进一步根据性别、吸烟状况及肺癌病理类型进行了分层分析。结果显示，无论性别或吸烟状态如何，摄入足量豆制品均能显著降低肺癌发生的风险（图4、5），在吸烟者中摄入足量豆制品的保护作用更为明显（HR=0.48, 95%CI: 0.30-0.76, P=1.73E-03）。此外，针对研究中部分有完整的疾病资料的肺癌病例进行了亚组分析（图6），腺癌患者占大多数，这与肺癌的总体流行病学特征一致。在腺癌患者中摄入足量豆制品同样显示肺癌发病风险显著降低（HR=0.64, 95%CI: 0.44-0.93, P=0.02），这与总体的研究结论一致；然而，由于鳞癌和小细胞肺癌的样本量较少，其结论的可靠性仍需进一步验证。

**图4:**
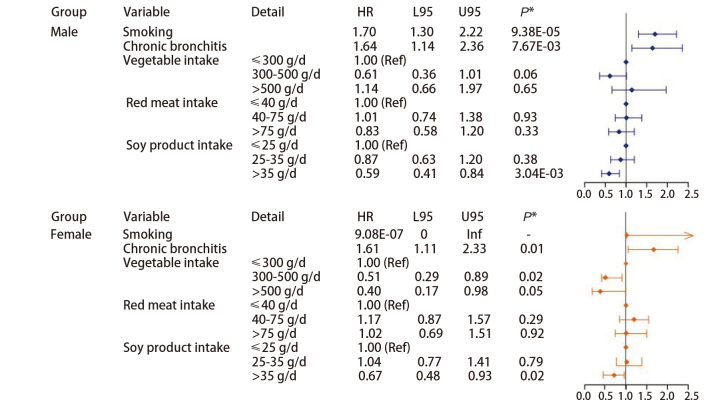
根据性别分层分析各变量与肺癌之间的关联

**图5:**
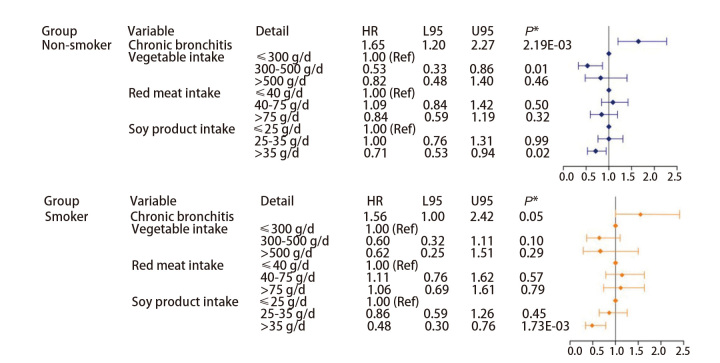
根据吸烟状况分层分析各变量与肺癌之间的关联

**图6:**
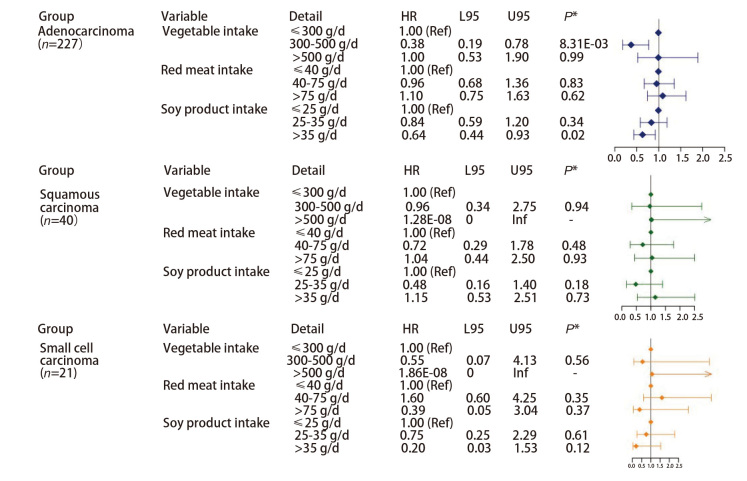
根据病理类型分层分析各变量与肺癌之间的关联

为了验证该结论在不同人群中的普适性，在UKB人群中进行了多因素分析，结果（表5）显示，在英国人群中尽管豆制品与肺癌发病的关系未达到统计学差异，但仍显示出潜在的保护趋势（HR=0.76, 95%CI: 0.55-1.06, P_adj_=0.10）。

**表5:** UKB人群中各变量与肺癌发病风险的关联

Variables	Detail	n (lung cancer)/n (all)	HR	95%CI	P*
BMI	<18.5 kg/m^2^	5/870	0.90	0.37-2.17	0.81
	18.5-24.0 kg/m^2^	285/58,057	1.00 (Ref)		
	24.1-28.0 kg/m^2^	384/67,924	1.00	0.85-1.16	0.96
	>28.0 kg/m^2^	217/33,715	1.12	0.93-1.33	0.23
Smoking	No	677/92,529	1.00 (Ref)		
	Yes	214/68,037	4.35	3.94-4.79	<2.00E-16
Chronic bronchitis	No	887/159,810	1.00 (Ref)		
	Yes	4/756	0.70	0.26-1.88	0.48
Vegetable intake	min-Q1	437/71,098	1.00 (Ref)		
	Q1-Q2	203/39,213	0.89	0.75-1.05	0.15
	Q3-max	251/50,255	0.79	0.68-0.93	3.81E-03
Red meat intake	min-Q1	53/12,950	1.00 (Ref)		
	Q2-Q3	378/72,669	0.97	0.73-1.31	0.86
	Q3-max	460/74,947	1.05	0.78-1.40	0.76
Soy products intake	0 g/d	847/149,601	1.00 (Ref)		
	0-35 g/d	7/2131	0.62	0.29-1.30	0.20
	>35 g/d	37/8834	0.76	0.55-1.06	0.10

*Multivarible Cox proportional hazard regression.

### 2.3 基于PRS的遗传因素与肺癌发病的关联

为了探索遗传风险对肺癌发病的影响，对SSACB的新发肺癌病例进行巢式病例对照研究，将肺癌患者与非肿瘤人群进行1:1.5匹配后，共有1083例病例和对照被纳入研究，并进行了GWAS芯片检测。

基于图2的PRS构建方法，通过既往报道的GWAS研究和本研究人群总共汇总得到23个位点，这些位点在SSACB病例对照人群中的关联分析如表6所示。评分的计算公式为PRS=Σ(β_i_×G_i_)。进一步在SSACB人群中研究PRS与肺癌发病风险之间的关联。根据PRS的分布密度图（图7）所示，肺癌病例的PRS的中位数显著大于对照组（Median_LC_=0.010, Median_control_=0.007, P=2.04E-07）。根据PRS评分将研究对象十等分，并拟合一条直线（y=0.24x+0.61）后发现肺癌发病风险与PRS评分基本呈正相关（r²=0.64, P<2E-16），肺癌的发病风险随着PRS评分的增加而升高（图8）。其中，PRS得分最高组的肺癌发病风险显著提高，其比值比（odds ratio, OR）达到3.96（95%CI: 2.17-7.22, P=6.93E-06），表明该组的肺癌发病风险是最低PRS组的近4倍。在低PRS组中（如第2组和第3组），虽然发病风险呈上升趋势，但由于95%CI包含1，其与参照组之间的差异无统计学意义。随着PRS的增加，肺癌发病风险急剧上升，特别在高PRS组（如第9组和第10组）中，其肺癌发病风险与最低PRS组相比均有统计学差异。这一发现提示，高PRS组的肺癌发病风险显著增加，应当作为重点对象进行个性化筛查与预防。

**表6:** 在SSACB巢式病例对照人群中中国人群PRS评分的23个位点与肺癌的关联分析

SNPs	CHR	Position	Effect allele	Reference allele	OR	P
Novel associations						
rs201871562	16	17266892	TAACA	T	0.88	0.278
rs752482	16	17271327	C	T	1.23	0.067
rs2041792	16	53405716	C	T	1.05	0.639
rs12602531	17	16757028	G	C	0.95	0.564
rs11657871	17	16762147	G	A	0.93	0.474
rs200995524	17	5081480	AC	A	1.01	0.960
rs4675932	2	242495223	T	C	0.87	0.355
rs6849419	4	102594545	A	G	0.90	0.329
rs9280834	6	29959057	TG	T	0.76	0.017
rs9261094	6	29970839	C	T	0.63	0.120
rs117287535	8	124250500	A	G	0.97	0.871
Previously reported associations						
rs7726159	5	1282319	A	C	1.29	4.28E-3
rs9374663	6	117782634	G	A	0.81	0.014
rs77468143	15	49376624	G	T	0.77	0.019
rs11928222	3	189350265	G	T	1.19	0.049
rs17038564	2	65496058	G	A	0.91	0.430
rs2293607	3	169482335	T	C	1.09	0.370
rs401681	5	1322087	T	C	0.93	0.420
rs3817963	6	32368087	C	T	1.04	0.719
rs1853837	6	41497035	A	C	1.16	0.116
rs4236709	8	32410110	G	A	0.87	0.246
rs4573350	9	124955115	T	C	1.03	0.757
rs10429489	9	21787521	A	G	0.86	0.137

CHR: chromosome; OR: odds ratio.

**图7:**
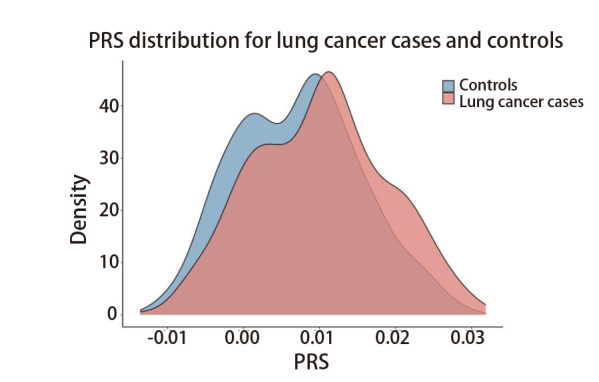
SSACB队列中肺癌病例及对照人群多基因风险评分分布密度图

**图8:**
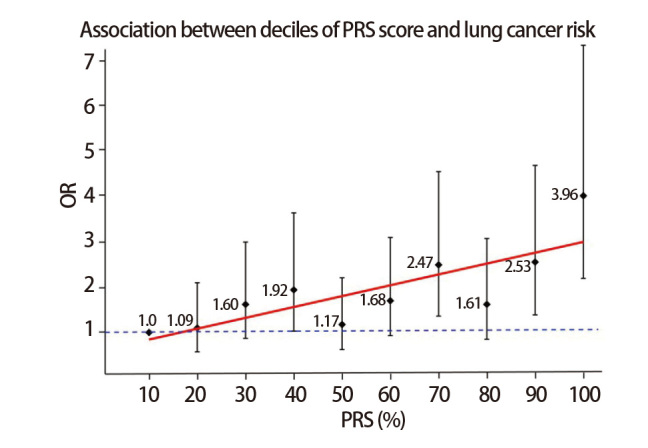
PRS评分十等分与肺癌发病风险的关联

随后通过3个不同的模型对PRS和肺癌的发病风险进行评估，经条件Logistic回归分析（表7）发现高PRS评分与肺癌发病有显著关联，模型3中加入豆制品摄入后一定程度上削弱了PRS与肺癌之间的关联（模型2：OR=1.92，95%CI: 1.32-2.79 vs 模型3：OR=1.88，95%CI: 1.26-2.80），提示饮食因素在遗传与肺癌的因果关系中可能起到一定效应修饰的作用。

**表7:** 低、中、高PRS评分与肺癌发生的关联

Model	Level of genetic risk	OR	95%CI	P
Model 1	Low	1.00 (Ref)		
	Intermediate	1.28	0.93-1.76	0.14
	High	2.03	1.47-2.80	1.73E-05
Model 2	Low	1.00 (Ref)		
	Intermediate	1.14	0.79-1.65	0.47
	High	1.92	1.32-2.79	6.07E-04
Model 3	Low	1.00 (Ref)		
	Intermediate	1.02	0.68-1.50	0.94
	High	1.88	1.26-2.80	1.84E-03

Model 1: Not adjusting any variables; Model 2: Adjusted for age, gender, smoking and chronic bronchitis; Model 3: Adjusted for age, gender, smoking, chronic bronchitis and soy product intake.

## 3 讨论

本研究基于一项中国上海地区人群前瞻性队列，发现摄入足量豆制品能够降低肺癌发生的风险，该结论在男性、女性、吸烟及从不吸烟者群体中均保持一致。同时，遗传因素也与肺癌发病密切关联，其风险随着肺癌PRS的增加而增加，且呈剂量-反应关系。

大豆作为豆制品的主要来源，富含异黄酮、磷脂等多种具有营养价值的植物蛋白质，且在制作工艺去除了大豆中影响蛋白吸收的物质，如胰蛋白酶抑制剂、植酸等，大大提升了豆制品的营养价值。基于中国和美国亚裔人群的病例对照研究，以及日本人群的前瞻性研究^[16]^显示，豆制品摄入量与多种癌症风险呈负相关，其中报道较多的是对乳腺癌和前列腺癌的保护作用，对肺癌的研究主要集中于非吸烟女性人群^[17]^。大豆异黄酮被认为是豆制品产生生物学作用的主要成分^[18]^，而金雀异黄酮又是其中含量最丰富、活性最强的异黄酮，是异黄酮发挥其生物学效应的基础^[19]^。研究^[20]^证明，金雀异黄酮能够抑制核因子κB（nuclear factor kappa-B, NF-κB）、磷脂酰肌醇3-激酶/蛋白激酶B（phosphoinositide 3-kinase/protein kinase B, PI3K/AKT）、转化生长因子β（transforming growth factor beta, TGF-β）等多种细胞增殖相关的信号通路，具有抑制癌细胞生长和转移、抗肿瘤血管生成等作用。饮食中摄入金雀异黄酮，有助于激活蛋白激酶，下调促炎反应，从而发挥抗肿瘤的功能^[21]^。

值得注意的是，在UKB人群中豆制品摄入与肺癌发病风险虽未呈现统计学关联，但仍显示出潜在的保护趋势。本课题组推测，这种现象可能源于人群异质性和膳食习惯差异。与中国人群相比，西方饮食中豆制品摄入量普遍较低且种类单一，而中国人群更倾向于摄入发酵豆制品和高异黄酮含量的豆制品（如豆豉、酱油等），其加工方式可能影响活性成分的生物利用度。此外，为了与中国膳食指南^[14]^保持一致，本项研究将UKB人群的豆制品摄入阈值设定为35 g/d，这一标准可能并不适用于英国人群的饮食习惯，导致保护效应未能显现。人体摄入豆制品后，在肠道菌群分泌的β-葡萄糖苷酶和宿主遗传因素共同作用下能够实现大豆异黄酮的体内代谢^[22]^。在亚洲人群中有40%-70%的个体能将大豆苷元代谢成雌马酚，而西方人群只有20%-30%^[23]^，这可能是此次研究中所观测到的豆制品在不同种族人群中效应差异的原因之一。

雌马酚与雌激素的结构功能相似，能与雌激素受体α和β结合，具有较强的类雌激素效应和抗氧化生物学活性，这可能是豆制品发挥抗癌功能的途径之一。现有证据^[24,25]^表明雌马酚可以通过抑制丝裂原活化蛋白激酶（mitogen-activated protein kinase, MAPK）信号通路诱导细胞转化中重要转录因子AP-1的激活而发挥抗肿瘤作用。相比大豆苷元，雌马酚因其亲脂性特征表现出更优的肠道吸收率和更长的体内半衰期，其生物利用度受到几个大豆异黄酮代谢酶的调控，如β-葡萄糖醛酸苷酶（glucuronidase beta, GUSB）、硫酸转移酶（sulfotransferase family 1A member 1, SULT1A1）、SULT2A1等，而对这些酶编码的基因（GUSB、SULT1A1和SULT2A1）通过调控酶的表达，也将间接影响雌马酚的生物可及性和最终生物转化效率。研究^[26]^表明，SULT1A1 Arg213His等位基因变异与肺癌发生风险增加有关，本课题组推测，与之相关的基因变异可能通过影响雌马酚的代谢合成能力，进而导致大豆异黄酮抗癌效应的个体差异。这种基因-营养互作机制可能为解释不同种族人群在豆制品抗癌效应的差异提供分子生物学依据。

本研究还利用PRS探讨了肺癌遗传因素在肺癌中的作用。PRS通过整合多个单核苷酸多态性（single nucleotide polymorphisms, SNPs）的累积效应能够量化个体遗传易感性，显著提高疾病预测的准确性^[27]^。与单一SNP位点相比，PRS更能捕捉复杂疾病的遗传异质性，全面反映遗传风险的累积作用。例如，Dai等^[15]^的研究发现PRS可以较为精准地评估肺癌的遗传风险，Lebrett等^[28]^也指出将PRS加入风险预测模型可有效提高肺癌筛查的准确性。基于中国人群的基因数据，本研究构建了具有全新位点的PRS模型，发现肺癌发病风险与PRS评分呈显著正相关，该结果与既往研究一致，进一步证实了遗传因素在肺癌发生发展过程中的关键作用。然而，PRS的应用仍存在一定局限性：首先，构建PRS模型时并不考虑单一SNP位点的遗传功能，这限制了我们对于遗传变异的致病机制理解^[29]^；其次，不同种族人群的遗传背景差异导致PRS模型的预测效能存在群体异质性。因此，未来的研究应加强遗传变异的功能注释，并开发适用于不同人群的优化PRS模型，以推动其从研究工具向临床应用的转化。

本研究的不足之处在于：（1）人群代表性受限，主要发现SSACB的研究对象主要来源于中国东南部郊区人群，该地区居民具有特定的社会经济背景，其膳食摄入模式可能更倾向于摄入蔬菜及豆制品，难以全面反映中国不同地域人群的饮食结构和遗传特征，存在一定选择偏倚。（2）混杂因素控制不足，研究中可能存在未测量的混杂变量，尤其是其他膳食成分的效应，例如研究未纳入的水果及全谷物摄入等，最终影响结论的准确性。（3）方法学及样本量的限制，首先，验证集UKB采用的膳食评估工具并非完整的食物频率问卷，可能存在信息偏倚；其次，SSACB队列中完成基因芯片检测的样本量有限，制约了遗传关联研究的统计效能，需在更大规模队列中进一步验证。

本研究的发现具有一定的预防疾病的公共卫生意义。通过对前瞻性队列人群的跟踪调查，发现高豆制品摄入量与发生肺癌的风险降低有关，并可在一定程度上降低高危人群的患癌风险。该结论对于肺癌高危人群的预防性饮食干预提供了一定的科学依据，同时也为制定适合中国人群特征的膳食指南和精准化预防策略奠定了理论基础。
